# Heterogeneous Activation of Signaling Pathways and Therapeutic Vulnerabilities in KSHV‐Associated Primary Effusion Lymphoma Cell Lines

**DOI:** 10.1002/jmv.70534

**Published:** 2025-08-02

**Authors:** Lianna Huang, Luping Chen, Yufei Huang, Shou‐Jiang Gao

**Affiliations:** ^1^ Cancer Virology Program, UPMC Hillman Cancer Center University of Pittsburgh School of Medicine Pittsburgh Pennsylvania USA; ^2^ Department of Microbiology and Molecular Genetics University of Pittsburgh School of Medicine Pittsburgh Pennsylvania USA; ^3^ Department of Medicine University of Pittsburgh School of Medicine Pittsburgh Pennsylvania USA

**Keywords:** AKT, dual inhibition, FOXOs, heterogeneity, Kaposi's sarcoma‐associated herpesvirus (KSHV), mTORC1, NF‐κB, PI3K, primary effusion lymphoma (PEL), therapy

## Abstract

Primary effusion lymphoma (PEL) is a rare and aggressive B‐cell malignancy caused by Kaposi's sarcoma‐associated herpesvirus (KSHV), with limited treatment options and poor prognosis. KSHV‐encoded proteins and miRNAs activate multiple signaling pathways that promote cell proliferation and survival. However, the heterogeneity in pathway activation and therapeutic responses among PEL cases remains poorly characterized. In this study, we investigated the activation status of key oncogenic and survival signaling pathways, including PI3K/AKT/mTOR, FOXOs and NF‐κB, and assessed the efficacy of targeted inhibitors in three KSHV‐positive EBV‐negative PEL cell lines BC3, BCP1 and BCBL1, KSHV‐negative BJAB cells, and KSHV‐infected BJAB‐KSHV cells. We observed heterogeneous activation of these pathways among PEL cell lines and differential sensitivity to pathway‐specific inhibitors. All KSHV‐infected cell lines exhibited constitutive AKT and mTORC1 activation and were sensitive to their respective inhibitors, though with varying efficacy. FOXO1 and FOXO3a, downstream targets of AKT, were frequently downregulated or inactivated by phosphorylation, consistent with AKT hyperactivation. Inhibition of FOXO1 suppressed proliferation and induced apoptosis in a cell line‐specific manner. Canonical and noncanonical NF‐κB pathways were differentially activated, and contributed to cell survival, as pathway‐specific inhibition suppressed proliferation. Interestingly, responses to inhibitors did not always correlate with basal pathway activation levels, highlighting the complexity of PEL signaling networks. Importantly, dual PI3K/mTOR inhibitors BGT226 and Dactolisib demonstrated superior efficacy by potently inhibiting proliferation and inducing apoptosis and cell cycle arrest in all PEL cell lines, suggesting an advantage in overcoming compensatory feedback mechanisms. These findings underscore the heterogeneity of PEL and the need for personalized therapeutic strategies. Our results support the potential of combinatorial or multi‐targeted approaches to improve treatment outcomes for PEL patients and warrant further preclinical and clinical investigations.

## 1 Introduction

Primary effusion lymphoma (PEL) is a rare and aggressive B‐cell lymphoproliferative disorder caused by Kaposi's sarcoma‐associated herpesvirus (KSHV) infection of precursor B cells [1]. It typically presents as lymphomatous effusions within body cavities, without detectable solid tumor masses, although dissemination to visceral organs has been observed in advanced disease. PEL predominantly affects immunocompromised individuals, particularly those with HIV/AIDS. Current treatment options are limited, and prognosis remains poor, with a median survival time of 5–6 months [2].

PEL cells are uniformly infected with KSHV, which remains predominantly in the latent phase, although a minority of cells undergo spontaneous lytic replication [3]. During latency, KSHV expresses several genes critical for tumor cell survival and proliferation [4]. These include latency‐associated nuclear antigen (LANA, ORF73), viral cyclin (vCyclin, ORF72), viral FLICE‐like inhibitory protein (vFLIP, ORF71), and a cluster of viral microRNAs (miRNAs). LANA ensures the maintenance of the KSHV episome by promoting latent genome replication and faithful segregation during mitosis [5]. It also supports cell proliferation and survival, inhibits lytic reactivation, and evades host immune surveillance [4]. vCyclin activates cellular cyclin‐dependent kinases, particularly CDK6, to drive cell cycle progression [6]. vFLIP constitutively activates the NF‐κB signaling pathway, thereby inhibiting apoptosis and promoting cell survival [7]. Viral miRNAs further contribute to oncogenesis by regulating both viral and host gene expression to enhance proliferation and survival [8].

PEL cells are often coinfected with Epstein–Barr virus (EBV), although the functional role of EBV in PEL remains unclear [3]. Gene expression profiling studies indicate little or no EBV gene expression in PEL [9]. Moreover, EBV‐negative PEL cell lines show distinct transcriptional profiles, including higher expression of KSHV genes compared to EBV‐positive lines, suggesting a potential suppressive effect of EBV on KSHV gene expression [10]. Importantly, EBV‐negative PELs are at least as aggressive as their EBV‐positive counterparts, indicating that KSHV alone is sufficient to drive PEL oncogenesis [3].

To better understand the basis for therapeutic resistance and poor clinical outcomes in PEL, we examined the activation status of key oncogenic and survival signaling pathways, including mTORC1, AKT, PI3K, FOXO, and NF‐κB. Focusing on KSHV‐positive, EBV‐negative PEL cell lines BC3, BCP1 and BCBL1, which are among the most widely studied, we also included the Burkitt lymphoma BJAB cell line and its KSHV‐infected counterpart, BJAB‐KSHV, for comparative purposes [11].

Our results demonstrate that although these PEL cell lines share some commonly activated pathways, they also exhibit distinct patterns of signaling pathway activation. Treatment with pathway‐specific inhibitors produced variable responses across cell lines, and there was no consistent correlation between pathway activation and inhibitor sensitivity. Notably, dual inhibition of PI3K and mTOR proved more effective in suppressing proliferation and inducing apoptosis, even in cell lines resistant to single‐agent treatments. These findings suggest that combined targeting of multiple signaling pathways may represent a promising therapeutic strategy to improve clinical outcomes in PEL.

## 2 Materials and Methods

### 2.1 Cell Lines and Cell Culture

PEL cell lines BC3, BCP1, and BCBL1, along with BJAB and BJAB‐KSHV cells, were cultured in RPMI‐1640 medium supplemented with 10% fetal bovine serum (FBS) and 1% penicillin‐streptomycin (100 µg/mL). Cells were treated with the following inhibitors: PI3K inhibitors GDC0941 (Selleckchem, Cat. No. S1065) and HS173 (Selleckchem, Cat. No. S7356); mTOR inhibitors Torin 2 (Selleckchem, Cat. No. S2817) and Rapamycin (Selleckchem, Cat. No. S1039); FOXO1 inhibitor AS1842856 (Sigma‐Aldrich, Cat. No. 344355); AKT inhibitor MK2206 (Selleckchem, Cat. No. S1078); NF‐κB inhibitors Bay11‐7082 (Bay11) (Selleckchem, Cat. No. S2913) and JSH23 (Selleckchem, Cat. No. S7351); NF‐κB2 inhibitor SN52 (MCE, Cat. No. HY‐P3229); and dual PI3K/mTOR inhibitors BGT226 (Selleckchem, Cat. No. S2749) and Dactolisib (NVP‐BEZ235; Selleckchem, Cat. No. S1009). All inhibitors were dissolved in DMSO, except SN52, which was dissolved in nuclease‐free water (Cytiva, Cat. No. SH30538.02).

### 2.2 Cell Proliferation Assay

BC3, BCP1, BCBL1, BJAB, and BJAB‐KSHV cells were seeded at a density of 5 × 10⁵ cells/mL in 2 mL of culture medium per well in 6‐well plates. Cells were treated with the indicated concentrations of each inhibitor in three biological replicates. Viable cells were counted daily for 5 days using a hemocytometer under a phase‐contrast microscope.

### 2.3 Western‐Blotting Analysis

Cells were cultured and treated as described above, then harvested 2 days posttreatment. Protein samples were prepared in 1× Laemmli buffer and boiled at 95°C for 10 min. Equal amounts of protein were resolved by SDS‐PAGE and transferred to Amersham Protran Premium 0.2 µm nitrocellulose membranes (Cytiva, Cat. No. 10600004). Membranes were blocked in 5% skim milk for 1 h and incubated overnight at 4°C with rabbit primary antibodies against the following proteins: GAPDH (CST, Cat. No. 5174; 1:5000), AKT (CST, Cat. No. 4691S), phosphorylated AKT (pAKT‐T308) (CST, Cat. No. 2965L), 4EBP1 (CST, Cat. No. 96445), phosphorylated 4EBP1 (p4EBP1) (CST, Cat. No. 2855), S6K (CST, Cat. No. 9202S), phosphorylated S6K (pS6K/pp70) (CST, Cat. No. 9205L), FOXO1 (CST, Cat. No. 2880), FOXO3 (CST, Cat. No. 2497), phosphorylated FOXO1/FOXO3a (pFOXO1‐Thr24/FOXO3a‐Thr32) (CST, Cat. No. 9464), p65 (CST, Cat. No. 8242), phosphorylated p65 (pp65) (Ser536) (CST, Cat. No. 3033), p105/p50 (CST, Cat. No. 13586), and p100/p52 (CST, Cat. No. 4882). All antibodies were used at 1:1000 dilution. After washing, membranes were incubated for 1 h with HRP‐conjugated goat anti‐rabbit IgG secondary antibody at 1:5000 dilution (CST, Cat. No. 7074S). Signal detection was performed using Immobilon Crescendo HRP Substrate (EMD Millipore, Cat. No. WBLUR0500) for GAPDH and SuperSignal West Femto Maximum Sensitivity Substrate (Thermo Fisher, Cat. No. 34096) for all other proteins. Chemiluminescent signals were visualized using the ChemiDoc MP Imaging System (Bio‐Rad, Cat. No. 17001402), and images were analyzed using Image Lab Software (Bio‐Rad). The relative intensities of all the target bands were normalized to those of the loading control GAPDH.

### 2.4 Analysis of Cell Cycle and Apoptosis

For cell cycle analysis, cells treated with inhibitors at the indicated concentrations for 2 days were labeled with 10 µM BrdU (Sigma‐Aldrich, Cat. No. B5002) for 2 h, then harvested and fixed in 70% ethanol. Cells were permeabilized with 2 M HCl, neutralized with 0.1 M sodium borate, and stained with an anti‐BrdU monoclonal antibody (Invitrogen, Cat. No. B35129) and propidium iodide (Sigma‐Aldrich, Cat. No. P4864). For apoptosis analysis, cells treated for 2 days were double‐stained with Fixable Viability Dye eFluor 660 (Invitrogen, Cat. No. 650864) and Annexin V PE‐Cy7 from the Annexin V Apoptosis Detection Kit (Invitrogen, Cat. No. 88810374). Flow cytometry was conducted using a CytoFLEX flow cytometer and CytExpert software (Beckman Coulter). Data analysis was performed using FlowJo v10 (BD Biosciences).

### 2.5 Statistical Analysis

Statistical analysis was performed using GraphPad Prism software. Results are presented as mean ± standard error of the mean (SEM). Group comparisons were analyzed by two‐way analysis of variance (ANOVA). All tests were two‐tailed, and a *p*‐value < 0.05 was considered statistically significant. The following symbols denote statistical significance: *p < 0.05, **p < 0.01, ***p < 0.001, ****p < 0.0001; “ns” indicates “not significant.”

## 3 Results

### 3.1 PEL Cell Lines Exhibit Heterogenous Activation of Signaling Pathways Involved in Cell Proliferation and Survival

The poor prognosis and chemoresistance associated with PEL may be attributable to the activation of signaling pathways that regulate cell proliferation and survival. To evaluate basal activation of such pathways, we performed Western‐blotting analysis of mTORC1, PI3K/AKT, FOXO, and NF‐κB signaling in the PEL cell lines BC3, BCP1, and BCBL1. BJAB and BJAB‐KSHV cells were included as reference controls, given the absence of matched normal counterparts for PEL cell lines.

**Figure 1:**
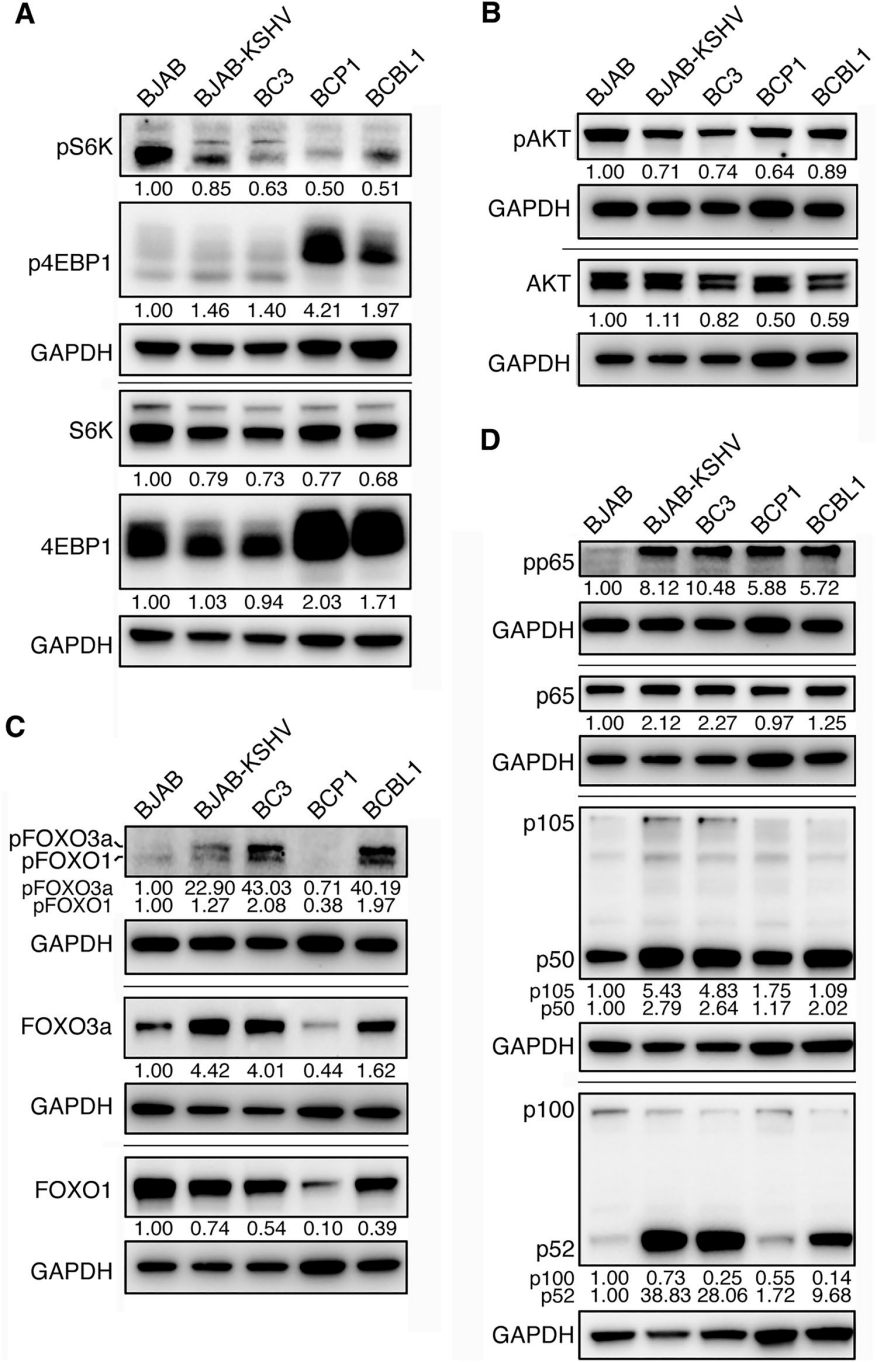
Western‐blotting analysis of activation of signaling pathways involved in cell proliferation and survival. (A) KSHV‐infected cell lines have constitutively activated but reduced pS6K and S6K levels compared to BJAB cells. BCP1 and BCBL1 cells had elevated 4EBP1 and p4EBP1 levels with slightly higher molecular weights for p4EBP1. (B) AKT was consistently activated in all PEL cell lines. (C) All the KSHV‐infected cells had lower levels of FOXO1 compared to BJAB cells. However, all the KSHV‐infected cells except BCP1 cells had higher levels of pFOXO1 compared to BJAB cells. BJAB‐KSHV, BC3, and BCBL1 cells had higher levels of FOXO3a and pFOXO3a while BCP1 cells had lower levels of FOXO3a and pFOXO3a compared to BJAB cells. (D) All the KSHV‐infected cells had strong constitutive activation of p65, and higher levels of p50 and p52 compared to BJAB cells. Samples were prepared 2 days after seeding. GAPDH was measured as an internal control. Experiments were independently repeated at least three times and representative results were presented.

We assessed mTORC1 activity by analyzing total and phosphorylated forms of its downstream targets, S6K and 4EBP1. KSHV ORF45 and miRNAs have been shown to activate the mTORC1 pathway [12, 13]. Total S6K levels were similar across all cell lines. However, total 4EBP1 levels were elevated in BCP1 cells by 2.03‐fold and BCBL1 cells by 1.71‐fold relative to BJAB cells (Figure 1A). All cells exhibited constitutive activation of phosphorylation of S6K (pS6K) and 4EBP1 (p4EBP1). Notably, stronger p4EBP1 levels and slower migration patterns, indicative of hyperphosphorylation, were more pronounced in BCP1 cells by 4.21‐fold and BCBL1 cells by 1.97‐fold compared to BJAB cells, suggesting stronger mTORC1 activation [14, 15].

AKT, a key upstream activator of mTORC1, is central to cell survival, proliferation and chemoresistance, and is activated downstream of PI3K [16]. Numerous KSHV proteins and miRNAs modulate AKT activity [17, 18, 19, 20]. Although pAKT was constitutively present in all tested cell lines, we observed minimal variations in total and pAKT levels across them (Figure 1B).

The FOXO family of transcription factors are downstream AKT targets. While FOXOs often act as tumor suppressors by promoting apoptosis and oxidative stress responses, they may also promote tumorigenesis via proangiogenic and pro‐metastatic effects [21, 22, 23]. Specifically, FOXO1 and FOXO3a have context‐dependent oncogenic or tumor‐suppressive roles. Their activity is negatively regulated by AKT‐mediated phosphorylation, leading to cytoplasmic sequestration [21, 22, 23]. Previous studies have implicated both FOXO1 and FOXO3a in the survival of PEL and KSHV‐transformed primary mesenchymal stem cells (KMMs), as well as in maintenance of viral latency via suppression of lytic replication [24, 25, 26]. However, phosphorylation status of FOXOs in PEL cells has not been previously characterized.

Our analysis revealed that compared to BJAB cells, total FOXO1 was markedly reduced in BJAB‐KSHV cells by 26%, BC3 cells by 46%, BCBL1 cells by 61%, and especially in BCP1 cells by 10‐fold lower (Figure 1C). Despite this, pFOXO1 was elevated in BC3 and BCBL1 cells, approximately twofold higher than in BJAB cells, suggesting enhanced inactivation and nuclear export. In BCP1 cells, pFOXO1 levels were only 38% of BJAB cells, likely due to extremely low total FOXO1. By contrast, total FOXO3a levels were significantly increased in BJAB‐KSHV cells by 4.42‐fold, BC3 cells by 4.01‐fold, and BCBL1 cells by 1.62‐fold, but were 56% lower in BCP1 cells. pFOXO3a, however, was dramatically elevated in all KSHV‐infected cells, 22.90‐fold in BJAB‐KSHV cells, 43.03‐fold in BC3 cells, and 40.19‐fold in BCBL1 cells, indicating pronounced inactivation of FOXO3a (Figure 1C). The pFOXO3a level in BCP1 cells was only 71% of BJAB cells, again could be due to the low total FOXO3a in these cells. Thus, FOXO1 and FOXO3a proteins are either downregulated or inactivated by phosphorylation in all KSHV‐infected cell lines.

KSHV activates both canonical and noncanonical NF‐κB pathways [7, 27, 28, 29]. Through interactions with the NF‐κB essential modulator (NEMO) and IKK complex, vFLIP induces phosphorylation and degradation of IκBα, resulting in NF‐κB activation [7, 28]. This promotes processing of p105 into p50 and phosphorylation of p65 (pp65), enabling nuclear translocation of p50‐p65 or p50‐p50 complexes and transcription of antiapoptotic genes [30]. Additionally, KSHV miRNAs target IκBα, further stimulating NF‐κB signaling [31, 32]. The noncanonical pathway, typically activated via TNF receptor family members, involves IKKα‐mediated phosphorylation of p100 and its processing into p52, which dimerizes with RelB to translocate into the nucleus [33]. This process is also enhanced by vFLIP [29].

BJAB‐KSHV and BC3 cells exhibited 2.12‐ and 2.27‐fold higher total p65, while BCP1 and BCBL1 cells showed minimal changes relative to BJAB cells (Figure 1D). All KSHV‐infected cells had significantly increased pp65 levels, ranging from 5.72‐ to 10.48‐fold higher, indicating strong canonical NF‐κB activation (Figure 1D). p105 and p50 levels were also elevated: BJAB‐KSHV and BC3 cells had 5.43‐ and 4.83‐fold increases in p105, and 2.79‐ and 2.64‐fold increases in p50, respectively. BCP1 cells showed a 1.75‐fold increase in p105 but unchanged p50, while BCBL1 cells had unchanged p105 but 2.02‐fold higher p50. Interestingly, p100 levels were reduced by 27%–86% in all KSHV‐infected lines, while p52 levels were markedly elevated by 38.83‐fold in BJAB‐KSHV cells, 28.06‐fold in BC3 cells, 9.68‐fold in BCBL1 cells, and 1.72‐fold in BCP1 cells, further confirming robust noncanonical NF‐κB activation, particularly in BJAB‐KSHV, BC3, and BCBL1 cells (Figure 1D).

Taken together, PEL cell lines exhibit heterogeneous but constitutive activation of multiple proliferation and survival pathways, including mTORC1, AKT, FOXOs, and NF‐κB. Given the availability of pharmacological inhibitors targeting these signaling cascades, some of which have shown promise in cancer treatment, we next investigated whether this heterogeneous signaling landscape correlates with differential responses to specific inhibitors.

### 3.2 PEL Cell Lines Exhibit Differential Sensitivities to mTORC1 Inhibitors Torin 2 and Rapamycin

The mTORC1 signaling pathway is a pro‐oncogenic axis frequently activated in cancer cells, including KS and PEL [34, 35, 36, 37]. We evaluated the efficacy of two mTORC1 inhibitors, Torin 2 and Rapamycin, on the PEL cell lines.

**Figure 2:**
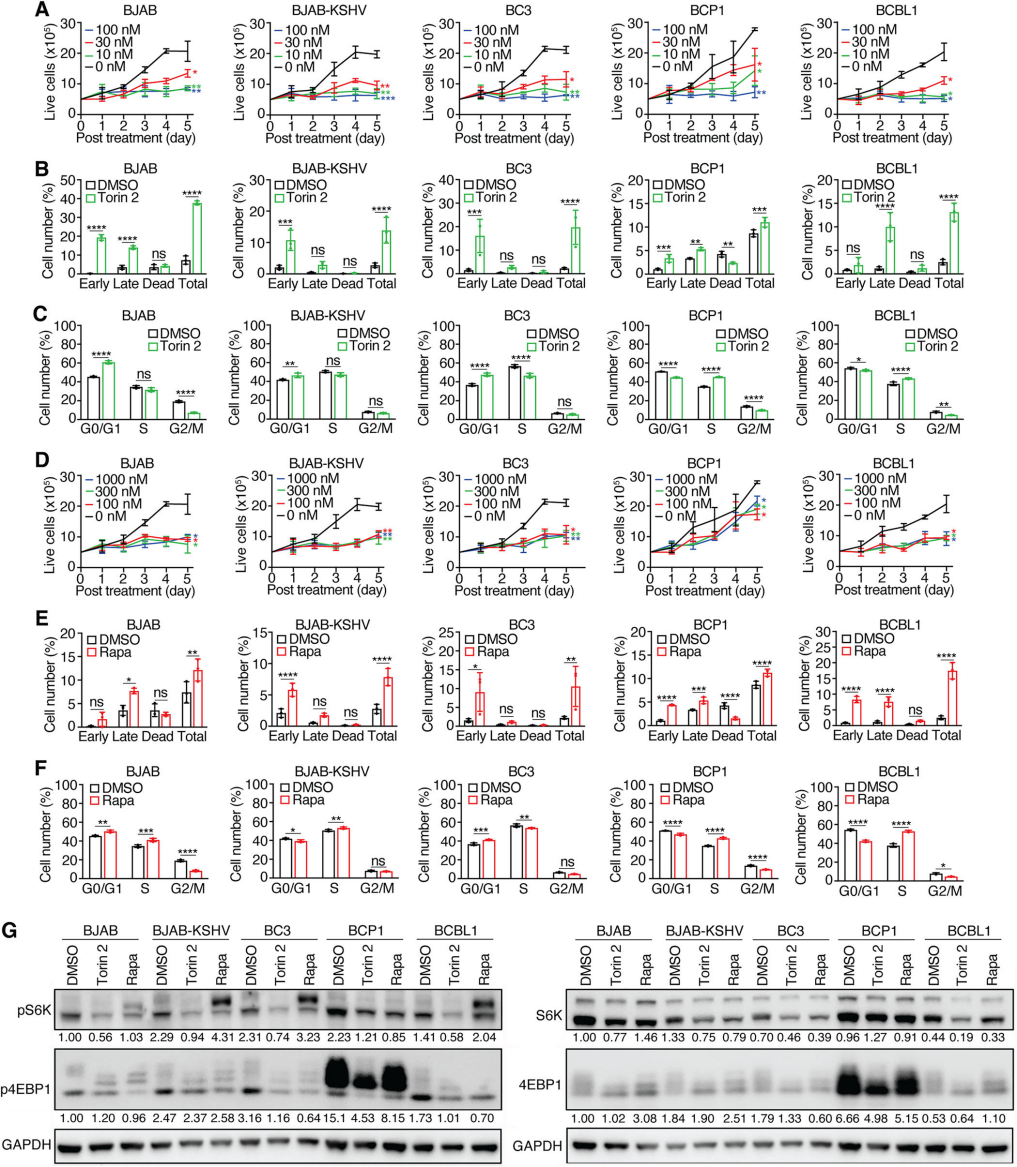
Effects of mTORC1 inhibitors Torin 2 and Rapamycin (Rapa) on PEL cell lines. (A) PEL cell lines were treated with different concentrations of Torin 2 and live cells were counted for 5 consecutive days. (B, C) PEL cell lines were analyzed for apoptotic and dead cells (B) and cell cycle progression (C) following treatment with 30 nM Torin 2 for 2 days. (D) PEL cell lines were treated with different concentrations of Rapamycin and live cells were counted for 5 consecutive days. (E, F) PEL cell lines were analyzed for apoptotic and dead cells (E) and cell cycle progression (F) following treatment with 300 nM Rapamycin for 2 days. (G) Western‐blotting analysis of mTORC1 downstream targets S6K and 4EBP1 for their total and phosphorylated levels in all the KSHV‐infected cell lines following treatment with 30 nM Torin 2 or 300 nM Rapamycin for 2 days. GAPDH was measured as an internal control. Experiments were independently repeated at least three times and representative results were presented.

Torin 2, an ATP‐competitive inhibitor of mTORC1 [38], inhibited the proliferation of all tested cell lines in a concentration‐dependent manner (Figure 2A). At 100 nM, Torin 2 nearly abrogated proliferation in all tested cell lines. However, the response at lower doses varied substantially. The IC_50_ values ranged from 3.5 nM in BJAB‐KSHV cells to 14.6 nM in BCP1 cells (Table 1). At 30 nM, Torin 2 reduced proliferation by 77%–95% in BJAB, BJAB‐KSHV, BC3, and BCBL1 cells but only inhibited BCP1 proliferation by 59% by Day 5 (Figure 2A).

**Table 1:** Summary of pathway activation and inhibitor effects on PEL cell lines.

mTORC1 pathway		BJAB	BJAB‐KSHV	BC3	BCP1	BCBL1
S6K (fold)		1.00	0.79	0.73	0.77	0.68
pS6K (fold)		1.00	0.85	0.63	0.50	0.51
pS6K (fold)		1.00	0.85	0.63	0.50	0.51
4EBP1 (fold)		1.00	1.03	0.94	2.03	1.71
p4EBP1 (fold)		1.00	1.46	1.40	4.21	1.97
Torin 2	IC_50_ (nM)	10.5	3.5	6.8	14.6	8.3
	Cell cycle arrest	G0/G1	G0/G1	G0/G1	S	S
	Apoptotic/dead cells (%)	38	14	20	11	13
Rapamycin	IC_50_ (nM)	22.1	60.1	57.9	> 1000	15.6
	Cell cycle arrest	G0/G1	S	G0/G1	S	S
		S				
	Apoptotic/dead cells (%)	12	8	11	11	17
AKT pathway		BJAB	BJAB‐KSHV	BC3	BCP1	BCBL1
AKT (fold)		1.00	1.11	0.82	0.50	0.59
pAKT (fold)		1.00	0.71	0.74	0.64	0.89
MK2206	IC_50_ (µM)	0.8	0.5	0.6	3.9	1.2
	Cell cycle arrest	G0/G1	G0/G1	G0/G1	G0/G1	G0/G1
	Apoptotic/dead cells (%)	36	9	13	14	20
GDC0941 (PI3K inhibitor)	IC_50_ (nM)	779	114	121.2	> 1000	159
	Cell cycle arrest	S	G0/G1	G0/G1	S	NC
	Apoptotic/dead cells (%)	23	10	34	9	21
HS173	IC_50_ (nM)	300	403	436	129	590
(PI3K inhibitor)	Cell cycle arrest	S	G0/G1	G0/G1	S	G0/G1
		G2/M	G2/M	G2/M	G2/M	G2/M
	Apoptotic/dead cells (%)	35	15	17	35	30
FOXO pathway		BJAB	BJAB‐KSHV	BC3	BCP1	BCBL1
FOXO3a (fold)		1.00	4.42	4.01	0.44	1.62
pFOXO3a (fold)		1.00	22.90	43.03	4.71	40.19
FOXO1 (fold)		1.00	0.74	0.54	0.10	0.39
pFOXO1 (fold)		1.00	1.27	2.08	0.38	1.97
AS1842856	IC_50_ (µM)	3.2	4.9	9.1	2.4	12.9
	Cell cycle arrest	G2/M	G0/G1	G0/G1	G2/M	G2/M
			G2/M	G2/M		
	Apoptotic/dead cells (%)	22	10	8	19	9
NF‐κB pathway		BJAB	BJAB‐KSHV	BC3	BCP1	BCBL1
p65 (fold)		1.00	2.12	2.27	0.97	1.25
pp65 (fold)		1.00	8.12	10.48	5.88	5.72
p105 (fold)		1.00	5.43	4.83	1.75	1.09
p50 (fold)		1.00	2.79	2.64	1.17	2.02
p100 (fold)		1.00	0.73	0.25	0.55	0.14
p52 (fold)		1.00	38.83	28.06	1.72	9.68
Bay11	IC_50_ (µM)	3.4	3.7	4.2	3.6	2.1
	Cell cycle arrest	G2/M	G0/G1	G0/G1	S	G0/G1
			G2/M	G2/M		G2/M
	Apoptotic/dead cells (%)	14	5	7	25	18
JSH23	IC_50_ (µM)	21.4	27.5	42.6	9.6	29.4
	Cell cycle arrest	NC	G0/G1	G0/G1	G0/G1	S
			G2/M	G2/M		
	Apoptotic/dead cells (%)	8	9	17	11	49
SN52	IC_50_ (µM)	89.7	63.7	90.9	77.4	87.4
	Cell cycle arrest	G0/G1 G2/M	G0/G1 G2/M	G0/G1 G2/M	G0/G1 G2/M	G0/G1 G2/M
	Apoptotic/dead cells (%)	99	73	89	96	83
PI3K/mTOR dual inhibitors		BJAB	BJAB‐KSHV	BC3	BCP1	BCBL1
BGT226	IC_50_ (nM)	11.7	0.7	6.5	24.9	25.5
	Cell cycle arrest	G0/G1	G0/G1	G0/G1	G0/G1	G0/G1
	Apoptotic/dead cells (%)	18	20	18	30	16
Dactolisib	IC_50_ (nM)	18.4	16.5	24.9	47.5	38.5
	Cell cycle arrest	G0/G1	G0/G1	G0/G1	S	NC
	Apoptotic/dead cells (%)	12	16	24	19	13

Abbreviation: NC, no change.

Apoptosis assay revealed that 30 nM Torin 2 induced cell death in all lines, ranging from 11% in BCP1 cells to 38% in BJAB cells (Figure 2B). Notably, BJAB cells showed the highest apoptotic and dead cell rate, correlating with its elevated basal pS6K levels, whereas all KSHV‐infected lines, including BJAB‐KSHV cells, exhibited reduced apoptotic and dead cells (< 20%), suggesting KSHV‐associated resistance. Although untreated BCP1 cells had a higher baseline of apoptotic and dead cells (8%) than other KSHV+ lines (< 3%), Torin 2 increased apoptotic and dead cells in BCP1 cells to only 11%. This modest response coincided with persistent p4EBP1 levels following treatment, as shown by Western‐blotting analysis (Figure 2G), implicating potential p4EBP1 activity in mediating resistance.

Cell cycle analysis revealed Torin 2‐induced G0/G1 arrest in BJAB, BJAB‐KSHV, and BC3 cells, indicative of a block in G1‐S transition (Figure 2C). In contrast, BCP1 and BCBL1 cells accumulated in S phase, suggesting S‐phase arrest, possibly linked to their elevated p4EBP1 levels (Figure 1A).

Rapamycin, a classical mTORC1 inhibitor that binds FKBP12 to destabilize the mTOR complex [39], has been shown to be efficacious against PEL cell lines [37]. Rapamycin at 100 nM suppressed proliferation in BJAB, BJAB‐KSHV, BC3, and BCBL1 cells by 66%–83% (Figure 2D). However, BCP1 cells again showed resistance, with only a 30% decrease in proliferation at 1000 nM.

At 300 nM, Rapamycin induced 8%–17% apoptotic and dead cells across all lines (Figure 2E), with BCP1 cells remaining the least responsive. Rapamycin had minimal effects on the cell cycle of BJAB‐KSHV and BC3 cells but induced G0/G1 and S‐phase accumulation with concurrent G2/M depletion in BJAB cells (Figure 2F). Similar to Torin 2, Rapamycin caused S‐phase arrest in BCP1 and BCBL1 cells. Notably, pS6K levels increased in BJAB‐KSHV, BC3, and BCBL1 cells posttreatment, possibly due to feedback regulation, while BCP1 cells retained elevated p4EBP1 despite treatment (Figure 2G).

Collectively, these data demonstrate that while both mTORC1 inhibitors broadly suppress proliferation, PEL cell lines, particularly BCP1 cells, exhibit distinct resistance patterns, possibly mediated by persistent p4EBP1 signaling.

### 3.3 AKT Inhibitor MK2206 Has Differential Effects on PEL Cell Lines

**Figure 3:**
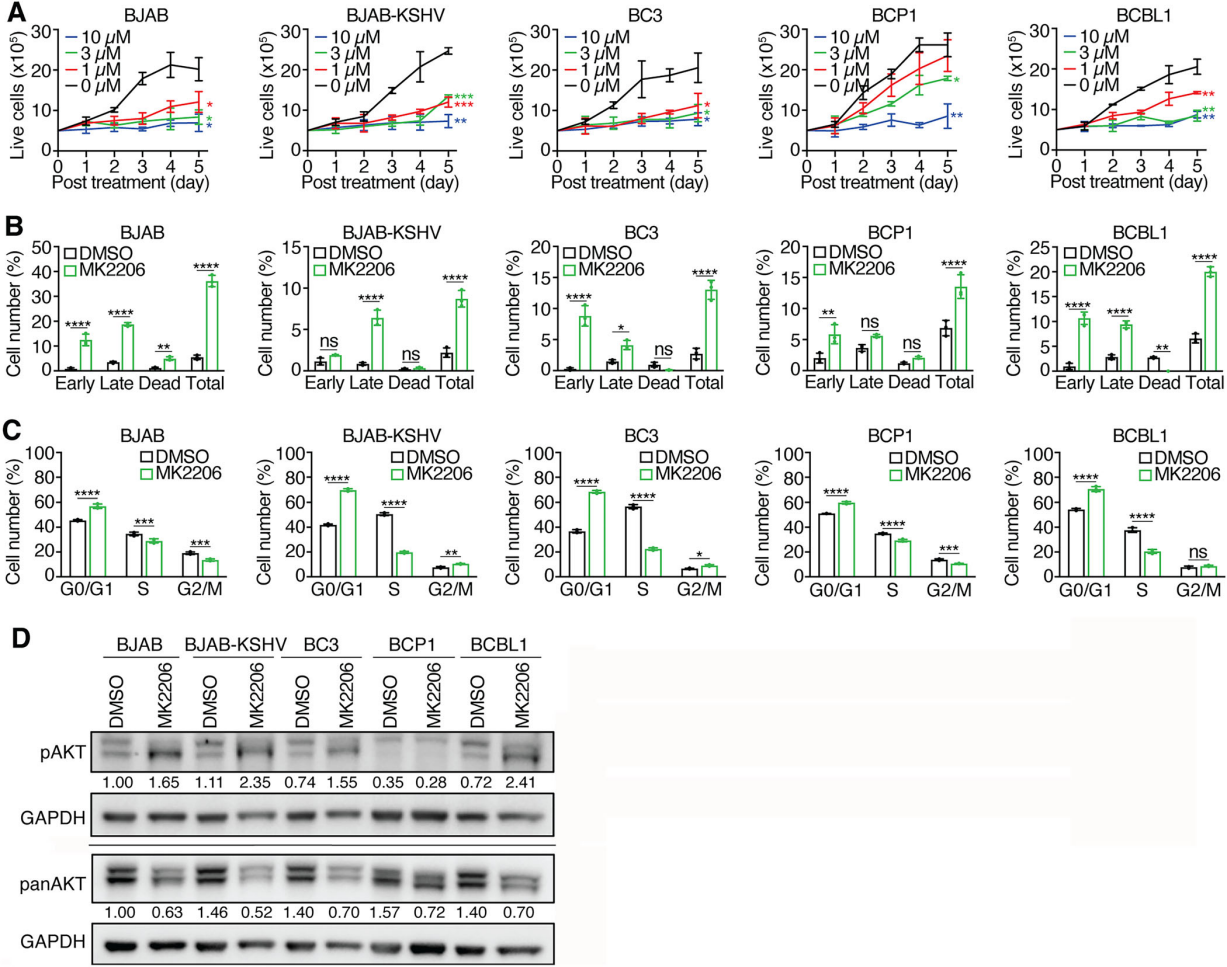
Effects of AKT inhibitor MK2206 on PEL cell lines. (A) PEL cell lines were treated with different concentrations of MK2206 and live cells were counted for 5 consecutive days. (B, C) PEL cell lines were analyzed for apoptotic and dead cells (B) and cell cycle progression (C) following treatment with 3 µM MK2206 for 2 days. (D) Western‐blotting analysis of total and phosphorylated AKT levels in all the KSHV‐infected cell lines following treatment with 3 µM MK2206 for 2 days. GAPDH was measured as an internal control. Experiments were independently repeated at least three times and representative results were presented.

Given the constitutive activation of AKT signaling in PEL cells, we evaluated MK2206, an allosteric AKT inhibitor [40], for its antiproliferative and proapoptotic effects. MK2206 inhibited the proliferation of all tested cell lines in a concentration‐dependent manner, with IC_50_ values ranging from 0.5 μM in BJAB‐KSHV cells to 3.9 μM in BCP1 cells (Figure 3A and Table 1). At 10 μM, MK2206 nearly completely blocked proliferation in all tested cell lines. At 3 μM, it reduced cell proliferation by > 75% in BC3 and BCBL1 cells but only by 40% in BCP1 cells.

Apoptotic and dead cells were significantly induced by MK2206 at 3 μM, ranging from 36% in BJAB cells to 9% in BJAB‐KSHV cells (Figure 3B and Table 1). In all cell lines, MK2206 also caused G0/G1 cell cycle arrest, with a concomitant decrease in S phase cells (Figure 3C), supporting a critical role of AKT in PEL cell cycle progression and survival.

Western‐blotting analysis revealed that MK2206 reduced total AKT protein across all cell lines (Figure 3D). Interestingly, MK2206 reduced the slower‐migrating pAKT band while increasing the faster‐migrating form in BJAB, BJAB‐KSHV, BC3, and BCBL1 cells, consistent with inhibition of AKT phosphorylation. However, no significant changes in pAKT were observed in BCP1 cells, further supporting their resistance profile.

### 3.4 PEL Cell Lines Exhibit Distinct Sensitivities to PI3K Inhibitors GDC0941 and HS173

To evaluate the therapeutic potential of PI3K pathway inhibition, we examined GDC0941, a pan‐PI3K inhibitor targeting all class I isoforms (p110α, δ, β, and γ) [41], and HS173, a PI3Kα‐selective inhibitor [42].

**Figure 4:**
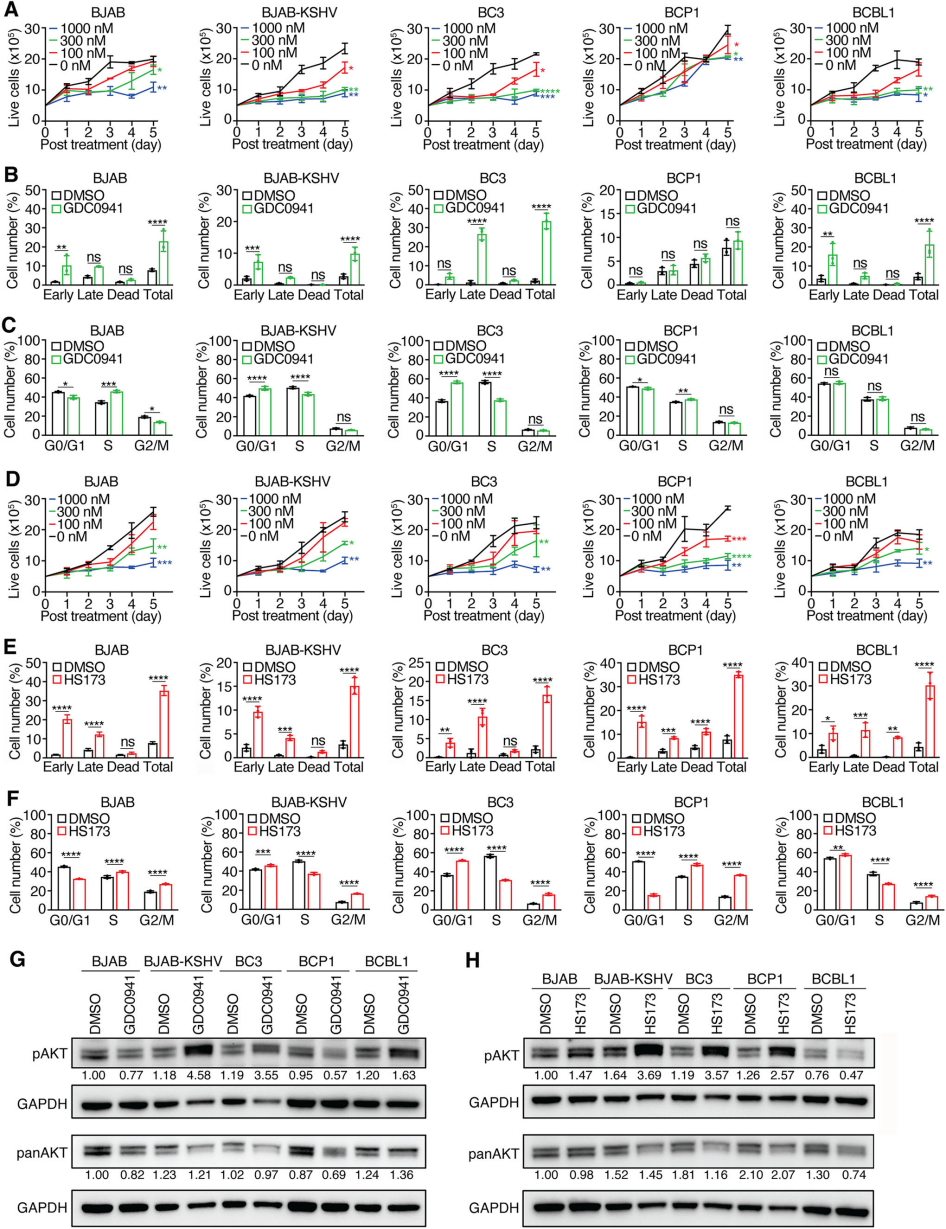
Effects of PI3K inhibitors GDC0941 and HS173 on PEL cell lines. (A) PEL cell lines were treated with different concentrations of GDC0941 and live cells were counted for 5 consecutive days. (B, C) PEL cell lines were analyzed for apoptotic and dead cells (B) and cell cycle progression (C) following treatment with 300 nM GDC0941 for 2 days. (D) PEL cell lines were treated with different concentrations of HS173 and live cells were counted for 5 consecutive days. (E, F) PEL cell lines were analyzed for apoptotic and dead cells (E) and cell cycle progression (F) following treatment with 300 nM HS173 for 2 days. (G, H) Western‐blotting analysis of total and phosphorylated AKT levels in all the KSHV‐infected cell lines following treatment with 300 nM GDC0941 (G) or HS173 (H) for 2 days. GAPDH was measured as an internal control. Experiments were independently repeated at least three times and representative results were presented.

GDC0941 inhibited the proliferation of all tested cell lines in a concentration‐dependent manner (Figure 4A), but BCP1 cells showed marked resistance. IC_50_ values ranged from 114 nM in BJAB‐KSHV cells to > 1000 nM in BCP1 cells (Table 1). At 1000 nM, BJAB‐KSHV, BC3, and BCBL1 proliferation was reduced by > 85%, whereas BCP1 cells were only inhibited by 35%. BJAB cells also exhibited partial resistance, with only 20% inhibition at 300 nM.

At 300 nM, GDC0941 induced apoptotic and dead cells in all cell lines except BCP1, ranging from 10% in BJAB‐KSHV cells to 32% in BC3 cells (Figure 4B and Table 1). G0/G1 arrest was observed in BJAB‐KSHV and BC3 cells, while BJAB and BCP1 cells showed weak S‐phase arrest (Figure 4C). No significant cell cycle changes occurred in BCBL1 cells. Interestingly, GDC0941 slightly reduced pAKT in BJAB and BCP1 cells but paradoxically increased pAKT in BJAB‐KSHV, BC3, and BCBL1 cells (Figure 4G), indicating potential compensatory feedback mechanisms commonly associated with PI3K inhibition [43].

HS173 suppressed proliferation in all tested cell lines with IC_50_ values ranging from 129 nM in BCP1 cells to 590 nM in BCBL1 cells (Figure 4D and Table 1). At 1000 nM, HS173 inhibited proliferation by > 70% in all tested cell lines. Notably, BCP1 cell lines, which were resistant to GDC0941, was the most sensitive to HS173.

Consistent with its growth‐inhibitory effects, HS173 at 300 nM induced apoptotic and dead cells in all tested cell lines, ranging from 15% in BJAB‐KSHV cells to 35% in BJAB and BCP1 cells (Figure 4E and Table 1). In addition, HS173 significantly induced G0/G1 cell cycle arrest in BJAB‐KSHV, BC3, and BCBL1 cells (Figure 4F). Western‐blotting analysis showed an increase in pAKT levels in all cell lines except BCBL1 cells following HS173 treatment, suggesting potential feedback activation of the PI3K‐AKT pathway in response to the inhibitor (Figure 4H).

Together, these results highlight the differential responses of PEL cell lines to various PI3K inhibitors and underscore the cell line‐specific variability in sensitivity and signaling responses to each inhibitor.

### 3.5 PEL Cell Lines Differentially Respond to FOXO1 Inhibitor AS1842856

Previous studies have demonstrated that FOXO1 is upregulated by KSHV miRNAs and vFLIP via the NF‐κB pathway, which maintains redox homeostasis and promotes KSHV‐induced proliferation and transformation [26]. In PEL cells, chemical or siRNA‐mediated inhibition of FOXO1 reactivates KSHV and increases apoptosis by inducing oxidative stress [25]. To investigate whether pharmacological inhibition of FOXO1 can inhibit PEL cells, we treated cells with AS1842856, a small molecule that specifically binds to and inhibits FOXO1 [44].

**Figure 5:**
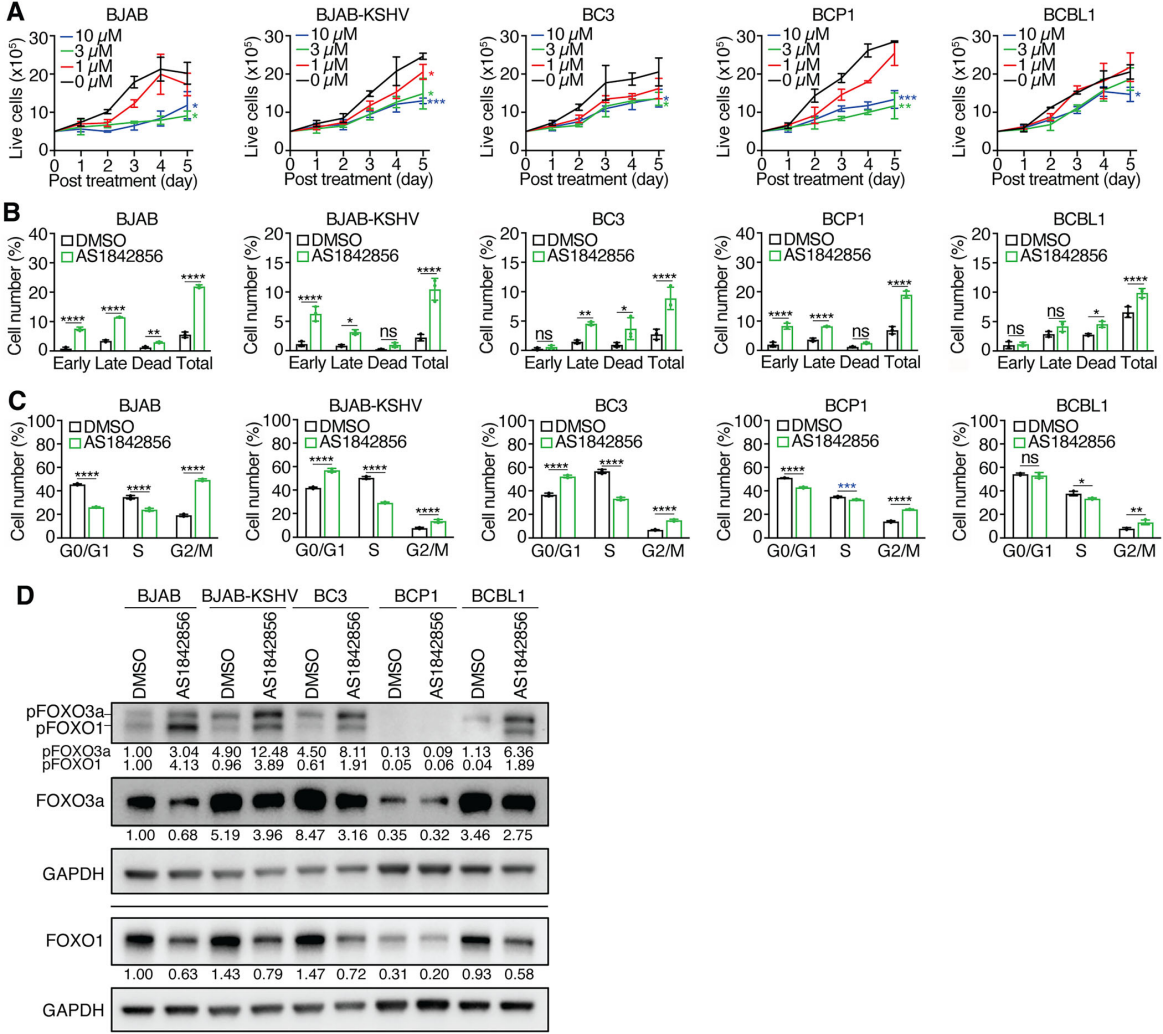
Effects of FOXO1 inhibitor AS1842856 on PEL cell lines. (A) PEL cell lines were treated with different concentrations of AS1842856 and live cells were counted for 5 consecutive days. (B, C) PEL cell lines were analyzed for apoptotic and dead cells (B) and cell cycle progression (C) following treatment with 3 µM AS1842856 for 2 days. (D) Western‐blotting analysis of total and phosphorylated FOXO1 and FOXO3a levels in all the KSHV‐infected cell lines following treatment with 3 µM AS1842856 for 2 days. GAPDH was measured as an internal control. Experiments were independently repeated at least three times and representative results were presented.

AS1842856 significantly suppressed proliferation in all tested cell lines (Figure 5A). However, BC3 and BCBL1 cells were less sensitive, with IC_50_ values of 9.1 and 12.9 µM, respectively, compared to 3.2, 4.9, and 2.4 µM in BJAB, BJAB‐KSHV, and BCP1 cells (Table 1). At 10 µM, AS1842856 inhibited BJAB, BJAB‐KSHV, and BCP1 proliferation by > 65% by Day 5, while inhibition in BC3 and BCBL1 cells was only 53% and 38%, respectively.

Apoptotic and dead cells were induced in all cell lines, ranging from 8% to 9% in BC3 and BCBL1 cells to 22% in BJAB cells (Figure 5B). AS1842856 induced G2/M arrest in all tested cell lines, and additionally caused G0/G1 arrest in BJAB‐KSHV and BC3 cells (Figure 5C).

Interestingly, AS1842856 reduced total FOXO1 and FOXO3a protein levels in all cell lines, but increased pFOXO1 and pFOXO3a levels, except in BCP1 cells (Figure 5D). The increase in phosphorylated FOXOs may reflect a feedback mechanism that triggers inactivation and nuclear export of FOXO proteins, leading to their subsequent proteasomal degradation [45].

### 3.6 Inhibitors of Canonical NF‐κB Pathway With Bay11 and JSH23 Effectively Suppress the Proliferation of PEL Cell Lines

**Figure 6:**
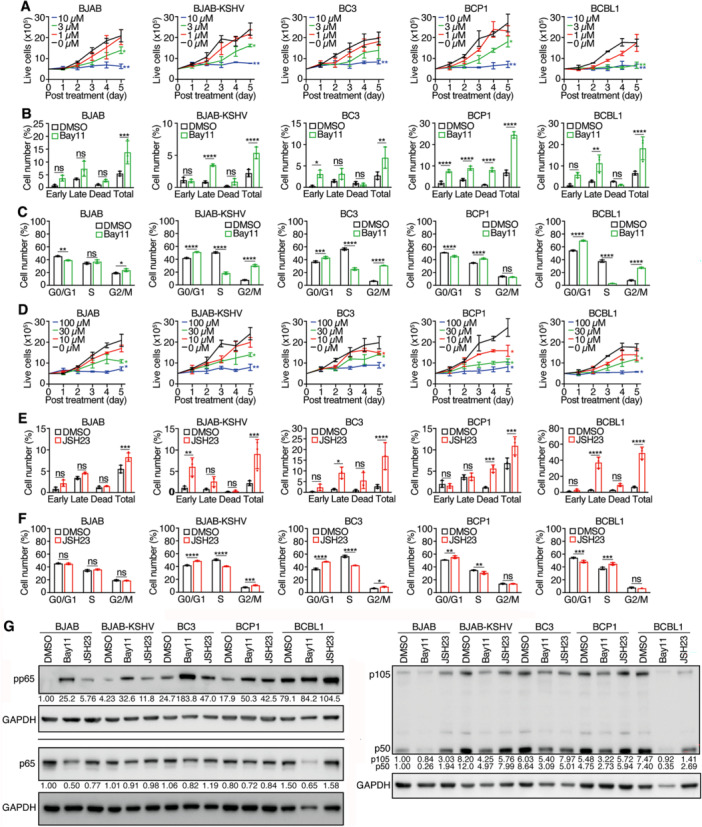
Effects of canonical NF‐κB inhibitors Bay11‐7082 (Bay11) and JSH23 on PEL cell lines. (A) PEL cell lines were treated with different concentrations of Bay11 and live cells were counted for 5 consecutive days. (B, C) PEL cell lines were analyzed for apoptotic and dead cells (B) and cell cycle progression (C) following treatment with 3 µM Bay11 for 2 days. (D) PEL cell lines were treated with different concentrations of JSH23 and live cells were counted for 5 consecutive days. (E, F) PEL cell lines were analyzed for apoptotic and dead cells (E) and cell cycle progression (F) following treatment with 30 nM JSH23 for 2 days. (G) Western‐blotting analysis of total and phosphorylated p65 levels in all the KSHV‐infected cell lines following treatment with 3 µM Bay11 or 30 nM JSH23 for 2 days. GAPDH was measured as an internal control. Experiments were independently repeated at least three times and representative results were presented.

Given the critical role of the canonical NF‐κB pathway in PEL cell survival [7, 28, 46], we evaluated the effects of two well‐characterized inhibitors: Bay11 and JSH23. Bay11, which blocks IκBα phosphorylation and prevents NF‐κB nuclear translocation [47], has been shown to be efficacious against PEL lines [28, 46]. Bay11 significantly inhibited proliferation in all tested cell lines in a dose‐dependent manner with IC_50_ values ranging from 2.1 to 4.2 µM (Figure 6A and Table 1). Despite BJAB cells exhibiting lower basal NF‐κB activity, they were sensitive to Bay11. At 10 µM, Bay11 suppressed > 90% proliferation in all tested cell lines by Day 5.

Bay11 at 3 µM significantly increased apoptotic and dead cells, with BJAB, BCP1 and BCBL1 cells reaching 14%, 25%, and 18%, respectively. BJAB‐KSHV and BC3 cells showed lower apoptotic and dead cell rates of 5% and 7% (Figure 6B and Table 1). Bay11 induced G0/G1 and G2/M arrest in BJAB‐KSHV, BC3, and BCBL1 cells, while BJAB cells showed weak G2/M arrest and BCP1 cells exhibited S‐phase arrest (Figure 6C). Western‐blotting analysis revealed increased pp65 in all cell lines, suggesting feedback activation of NF‐κB. Total p65 was reduced in BJAB and BCBL1 cells, while p105 and p50 were decreased across all lines, with the strongest reductions in BCP1 and BJAB cells (Figure 6G).

JSH23, which inhibits NF‐κB nuclear translocation without blocking IκBα degradation [48], also potently inhibited proliferation of all tested cell lines in a concentration‐dependent manner (Figure 6D). The IC_50_ values were 21.4 µM for BJAB cells, 27.5 µM for BJAB‐KSHV cells, and 29.4 µM for BCBL1 cells, with BC3 cells being less sensitive, having a IC_50_ value of 42.6 µM and BCP1 being more sensitive, having a IC_50_ value of 9.6 µM (Table 1). At 100 µM, JSH23 inhibited > 90% of proliferation in all tested cell lines.

JSH23 at 30 µM induced apoptotic and dead cells in all cell lines, most notably in BCBL1 cells at 49%, followed by 8%–17% in the other lines (Figure 6E and Table 1). It induced G0/G1 arrest in BJAB‐KSHV, BC3 and BCP1 cells, while BCBL1 cells exhibited S‐phase arrest and BJAB cells showed no significant cell cycle changes (Figure 6F). Similar to Bay11, JSH23 increased pp65 levels but did not alter total p65 level. It reduced p50 in BC3 and BCBL1 cells, with minimal effects on p105 and other cell lines (Figure 6G).

### 3.7 NF‐κB Noncanonical Inhibitor SN52 Inhibits the Proliferation of PEL Cells

**Figure 7:**
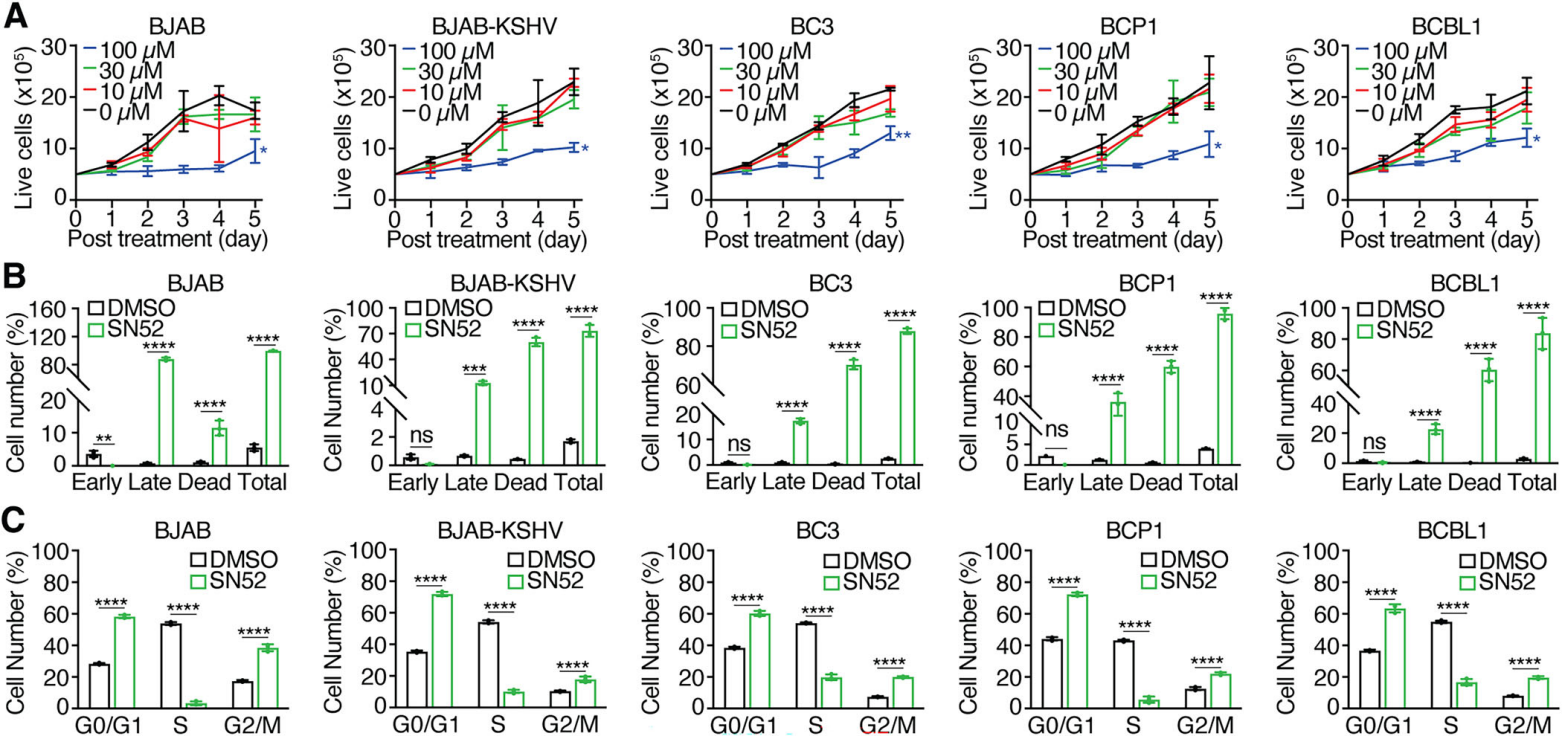
Effects of noncanonical NF‐κB inhibitor SN52 on PEL cell lines. (A) PEL cell lines were treated with different concentrations of SN52 and live cells were counted for 5 consecutive days. (B, C) PEL cell lines were analyzed for apoptotic and dead cells (B) and cell cycle progression (C) following treatment with 100 µM SN52 for 2 days. Experiments were independently repeated at least three times and representative results were presented.

To explore whether the noncanonical NF‐κB pathway also contributes to PEL cell survival, we treated the cells with SN52, which inhibits the nuclear translocation of the p52‐RelB complex [49]. At 100 µM, SN52 significantly suppressed cell proliferation across tested all lines, achieving > 55% inhibition by Day 5 (Figure 7A). However, at concentrations ≤ 30 µM, inhibition was minimal. Despite the initial potent cytotoxic effect at 100 µM (≥ 73% apoptotic and dead cells at Day 2, Figure 7B and Table 1), we observed resumed proliferation by Day 5, likely due to the compound's short half‐life, as the treatment was not replenished. SN52 induced both G0/G1 and G2/M arrest in all cell lines examined (Figure 7C). Unexpectedly, immunofluorescence staining and Western‐blotting analysis following cytoplasmic and nuclear fractionation failed to detect SN52 blockage of p52 nuclear translocation at 12 h or 48 h posttreatment (data not shown).

### 3.8 PI3K/mTOR Dual Inhibitors BGT226 and Dactolisib Inhibit the Proliferation of PEL Cells

Given the concurrent activation of PI3K and mTOR signaling in PEL cells, we tested the dual inhibitors BGT226 and Dactolisib, both of which target pan‐class PI3Ks and mTOR, and have been studied for their anticancer effects in clinical trials [50, 51]. Dactolisib has also been shown to efficacious against PEL cell lines [52].

**Figure 8:**
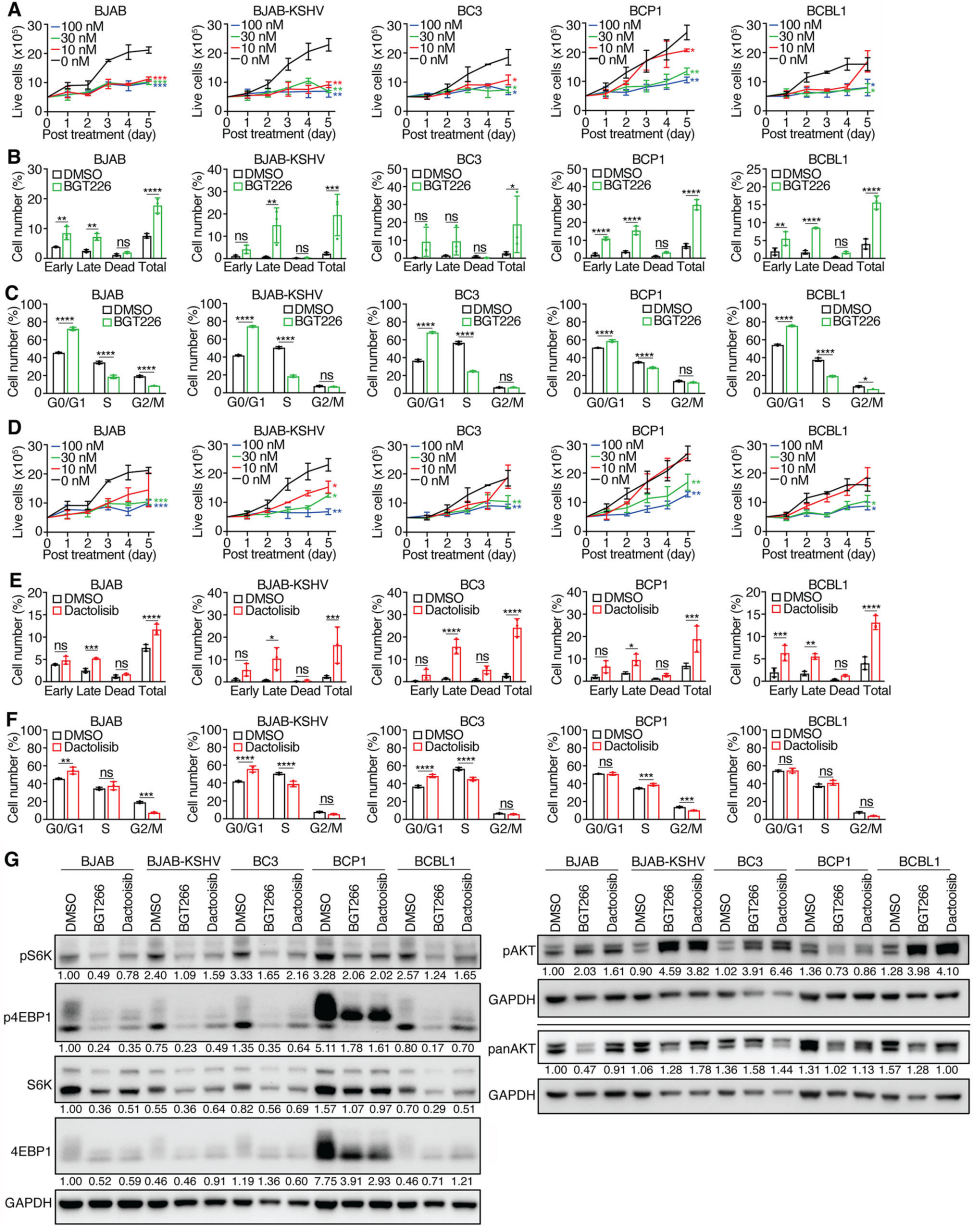
Effects of PI3K/mTOR dual inhibitors BGT226 and Dactolisib on PEL cell lines. (A) PEL cell lines were treated with different concentrations of BGT226 and live cells were counted for 5 consecutive days. (B, C) PEL cell lines were analyzed for apoptotic and dead cells (B) and cell cycle progression (C) following treatment with 30 nM BGT226 for 2 days. (D) PEL cell lines were treated with different concentrations of Dactolisib and live cells were counted for 5 consecutive days. (E, F) PEL cell lines were analyzed for apoptotic and dead cells (E) and cell cycle progression (F) following treatment with 30 nM Dactolisib for 2 days. (G) Western‐blotting analysis of PI3K downstream target AKT, and mTORC1 downstream targets S6K and 4EBP1 for their total and phosphorylated levels in all the KSHV‐infected cell lines following treatment with 30 nM BGT226 or Dactolisib for 2 days. GAPDH was measured as an internal control. Experiments were independently repeated at least three times and representative results were presented.

BGT226 significantly inhibited proliferation in all tested cell lines, especially at 100 nM where > 60% inhibition was observed by Day 5 (Figure 8A). At 10 nM, BJAB and BJAB‐KSHV cells were more sensitive, while BCP1 and BCBL1 cells responded weakly, which occurred 3 days after treatment. It is unclear whether these weak responses of BCP1 and BCBL1 cells to this dose were due to the short half‐life of the inhibitor or development of resistance as a result of a feedback mechanism. BGT226 induced apoptotic and dead cells in all cell lines, ranging from 16% in BCBL1 cells to 30% in BCP1 cells (Figure 8B and Table 1), and caused G0/G1 arrest in all cell lines (Figure 8C). It reduced pS6K and p4EBP1 levels in all cell lines and, in most cases, also reduced total levels of these proteins. However, BGT226 increased the levels of pAKT in all the cell lines examined except BCP1 cells (Figure 8G), possibly due to a feedback mechanism [43].

Dactolisib similarly inhibited cell proliferation in a dose‐dependent manner in all tested cell lines (Figure 8D). IC_50_ values ranged from 16.5 nM for BJAB‐KSHV cells to 47.5 nM for BCP1 cells (Table 1). At 100 nM, it inhibited > 65% of proliferation in all cell lines. Dactolisib induced apoptotic and dead cells (12%–24%) and G0/G1 arrest in BJAB, BJAB‐KSHV, and BC3 cells. In contrast, it induced weak G2/M arrest in BCP1 cells and had no effect on BCBL1 cell cycle progression (Figure 8F). Like BGT226, it reduced pS6K and p4EBP1 and increased pAKT levels in all cell lines (Figure 8G).

## 4 Discussion

In this study, we conducted a comprehensive analysis of pathway activation and pharmacological response to a panel of inhibitors in three KSHV‐positive EBV‐negative PEL cell lines including BC3, BCP1, and BCBL1, as well as a KSHV‐negative cell line BJAB and its KSHV‐infected derivative BJAB‐KSHV. Our results reveal substantial heterogeneity in pathway activation profiles across PEL lines and similarly diverse responses to pathway‐specific inhibitors. Notably, the basal activation levels of a pathway did not consistently predict the cell line's sensitivity to its respective inhibitor, highlighting the complexity of KSHV‐driven signaling networks and the need for personalized therapeutic strategies.

As expected, KSHV‐positive cell lines exhibited constitutive activation of the PI3K/AKT/mTOR axis, consistent with prior findings that multiple KSHV products, including vGPCR, vIL6, and viral miRNAs, can engage this pathway [17, 18, 19, 20]. All KSHV‐infected lines demonstrated AKT phosphorylation and downstream mTORC1 activation, as evidenced by S6K and 4EBP1 phosphorylation. BCP1 and BCBL1 cells, in particular, showed pronounced 4EBP1 hyperphosphorylation. Interestingly, FOXO1/3a phosphorylation, another AKT target, varied across lines with BC3, BJAB‐KSHV, and BCBL1 cells having higher levels, whereas BCP1 cells having lower total and phosphorylated levels. This suggests divergent FOXO regulation, possibly influenced by additional signaling or epigenetic mechanisms.

Inhibitors targeting AKT (MK2206), PI3K (GDC0941 and HS173), and mTORC1 (Rapamycin and Torin2) all reduced proliferation of PEL cell lines and induced apoptosis, cell death and cell cycle arrest. However, consistent with the pathway activation results, responses were cell line‐dependent. BCP1 cells, despite strong mTORC1 activation, were relatively resistant to Rapamycin and GDC0941 but sensitive to the PI3Kα‐specific inhibitor HS173, suggesting differential reliance on specific PI3K isoforms or feedback loops. Notably, while both Torin2 and Rapamycin target mTORC1, Torin2 also affects mTORC2 and had broader efficacy [53], underscoring its potential as a superior agent in this context.

The FOXO inhibitor AS1842856 also reduced proliferation across PEL lines, but again with variable efficacy. BC3 and BCBL1 cells were more resistant to the drug even at higher doses, contrasting with previous findings using lower concentrations [25]. This discrepancy could be attributed to differences in culture conditions, cell seeding density, or cell line adaptation, emphasizing the need for standardized protocols when comparing therapeutic responses.

NF‐κB signaling is another critical survival pathway in PEL cells. Constitutive activation of both canonical and noncanonical NF‐κB pathways was confirmed in all KSHV‐positive lines, driven primarily by vFLIP and viral miRNAs [7, 28, 29, 31, 32]. Pharmacological inhibition of the canonical NF‐κB pathway using Bay11 and JSH23 effectively suppressed cell proliferation and induced apoptosis and cell death, with some variation in affected cell cycle phases. The noncanonical pathway inhibitor SN52 also reduced proliferation and induced apoptosis and cell death but showed reduced efficacy in BC3 and BCBL1 cells, highlighting potential pathway rewiring or alternative compensatory mechanisms. While inhibitors of both canonical and noncanonical NF‐κB pathways can inhibit the proliferation of PEL cell lines, these inhibitors usually have systematic and on‐target toxicities, which limit their clinical usage [54, 55].

Importantly, despite activation of multiple signaling pathways across PEL cell lines, monotherapy with single‐pathway inhibitors yielded inconsistent and often incomplete responses. This suggests that redundancy and cross‐talk between pathways may limit the effectiveness of targeted monotherapies. To overcome this, we investigated dual PI3K/mTOR inhibitors (BGT226 and Dactolisib), which exhibited markedly enhanced potency across all PEL cell lines, even at low nanomolar concentrations. These agents robustly suppressed downstream mTOR targets and induced apoptosis, cell death and cell cycle arrest, effectively bypassing the variability seen with single inhibitors. Interestingly, despite reducing total AKT levels, both dual inhibitors increased pAKT, likely due to feedback activation upon mTOR inhibition, a phenomenon previously reported [56].

Our findings underscore several key points for future PEL treatment strategies. First, PEL is a molecularly heterogeneous disease, and pathway activation varies significantly among cell lines, even among those only harboring KSHV. Second, therapeutic sensitivity to pathway inhibitors cannot be reliably predicted by basal pathway activity alone, and functional validation remains essential. Third, combination strategies, whether using dual‐specificity inhibitors or cocktails of single‐target agents, may be necessary to achieve sustained suppression of compensatory signaling and prevent drug resistance. Dual PI3K/mTOR inhibitors emerge from our study as particularly promising candidates for further preclinical and translational development in PEL.

Future studies should aim to (1) dissect the underlying molecular determinants of pathway heterogeneity in PEL; (2) identify predictive biomarkers of therapeutic response; and (3) explore combinatorial therapies targeting complementary pathways or incorporating immune modulation. Examination of PEL cells freshly derived from patients may offer additional translational insights.

In summary, our results reveal extensive heterogeneity in signaling activation and drug sensitivity among PEL cell lines. While single‐pathway inhibitors demonstrate limited and variable efficacy, dual‐target inhibitors, particularly those acting on the PI3K/mTOR axis, exhibit superior antitumor activity. These findings provide a strong rationale for developing individualized, multitargeted therapeutic strategies to improve outcomes for patients with this otherwise intractable lymphoma.

## Author Contributions

Lianna Huang performed experiments, data analysis, and writing original draft. Luping Chen helped with the experiments and provided supervision of this study. Yufei Huang provided resources, and contributed to writing, review and editing. Shou‐Jiang Gao conceptualized this study, secured funding, provided resources and supervision, and contributed to writing, review and editing.

## Conflicts of Interest

The authors declare no conflicts of interest.

## Data Availability

The data that support the findings of this study are available on request from the corresponding author. The data are not publicly available due to privacy or ethical restrictions.
